# Activation of Type 4 Metabotropic Glutamate Receptor Regulates Proliferation and Neuronal Differentiation in a Cultured Rat Retinal Progenitor Cell Through the Suppression of the cAMP/PTEN/AKT Pathway

**DOI:** 10.3389/fnmol.2020.00141

**Published:** 2020-08-20

**Authors:** Zhichao Zhang, Yingfei Liu, Yan Luan, Kun Zhu, Baoqi Hu, Bo Ma, Li Chen, Xuan Liu, Haixia Lu, Xinlin Chen, Yong Liu, Xiaoyan Zheng

**Affiliations:** ^1^Institute of Neurobiology, Xi’an Jiaotong University Health Science Center, Xi’an, China; ^2^Department of Ophthalmology, The First Affiliated Hospital of Xi’an Jiaotong University, Xi’an, China; ^3^Department of Hematology, The First Affiliated Hospital of Xi’an Jiaotong University, Xi’an, China

**Keywords:** mGluR4, retinal progenitor cells, proliferation, differentiation, cAMP/PTEN/AKT pathway

## Abstract

Retinal progenitor cells (RPCs) remain in the eye throughout life and can be characterized by their ability for self-renewal as well as their specialization into different cell types. A recent study has suggested that metabotropic glutamate receptors (mGluRs) participate in the processes of multiple types of stem cells. Therefore, clarifying the functions of different subtypes of mGluRs in RPCs may provide a novel treatment strategy for regulating the proliferation and differentiation of endogenous RPCs after retinal degeneration. In this study, we observed that mGluR4 was functionally expressed in RPCs, with an effect on cell viability and intracellular cAMP concentration. The activation of mGluR4 by VU0155041 (VU, mGluR4 positive allosteric selective modulator) reduced the number of BrdU^+^/Pax6^+^ double-positive cells and Cyclin D1 expression levels while increasing the number of neuron-specific class III beta-tubulin (Tuj1)- and Doublecortin (DCX)-positive cells. The knockdown of mGluR4 by target-specific siRNA abolished the effects of VU on RPC proliferation and neuronal differentiation. Further investigation demonstrated that mGluR4 activation inhibited AKT phosphorylation and up-regulated PTEN protein expression. Moreover, the VU0155041-induced inhibition of proliferation and enhancement of neuronal differentiation in RPCs were significantly hampered by Forskolin (adenylyl cyclase activator) and VO-OHpic trihydrate (PTEN inhibitor). In contrast, the effect of LY294002 (a highly selective Akt inhibitor) on proliferation and differentiation was similar to that of VU. These results indicate that mGluR4 activation can suppress proliferation and promote the neural differentiation of cultured rat RPCs through the cAMP/PTEN/AKT pathway. Our research lays the foundation for further pharmacological work exploring a novel potential therapy for several retinal diseases.

## Introduction

Retinal progenitor cells (RPCs) originate in the embryonic neural ectoderm and remain in the ciliary body, at the retinal border in the adult. These multipotent progenitor cells can give rise to seven cell types, including retinal ganglion cells, horizontal cells, bipolar cells, amacrine cells, cone photoreceptors, rod photoreceptors, and Müller glia cells (Gariano and Gardner, 2005). Therefore, RPC replacement therapy promises to be a novel therapeutic strategy for retinal degeneration such as age-related macular degeneration (AMD), retina splitting disease, and retinitis pigmentosa (RP). However, the number of RPCs has limited their clinical application. Therefore, it is urgent to identify the underlying mechanisms that allow for their positive enrichment. Recent evidence has indicated that glutamate is involved in regulating the survival, proliferation, and differentiation of different types of stem cells (Uccelli, 2013; Naszai and Cordero, 2016; Reichenbach et al., 2018). These findings stir concern about glutamate and its receptors in the regulation of RPC behaviors.

The metabotropic glutamate receptors (mGluRs), including eight subtypes subdivided into three groups, belong to G-protein-coupled receptors (Gerber et al., 2007). As a member of group III mGluRs, the type 4 metabotropic glutamate receptor (mGluR4) is mainly located in presynaptic terminals and considered to mediate the presynaptic depression of glutamatergic synaptic transmission, which manifests as inhibiting glutamate release (Corti et al., 2002). Recent studies had indicated that mGluR4 participates in the process of cell proliferation, differentiation, and survival in several different cell types which include neural stem cells (NSCs), granule neuroprecursors, bladder cancer, glioma, etc. For example, our previous study showed that mGluR4 activation regulated proliferation, neuronal differentiation, and survival in rat NSCs (Zhang et al., 2015, 2020). In addition to NSCs, mGluR4 also inhibited cell proliferation and promoted cell apoptosis in both bladder cancer and glioma (Zhang et al., 2018, 2019). Beyond our own research, evidence from cerebellar granular neuroprecursors and embryonic stem cells also suggests that mGluR4 may be involved in promoting neural differentiation and inhibiting cell proliferation (Nakamichi et al., 2008). These results suggest that mGluR4 may act as a key receptor in regulating cell behavior. However, our understanding of the role of mGluR4 in the regulation of RPC proliferation and differentiation remains insufficient.

In this study, we focused on mGluR4 in the regulation of the proliferation and neural lineage commitment of RPCs. We observed that mGluR4 was functionally expressed in rat-cultured RPCs and that mGluR4 activation with VU0155041 (a positive allosteric selective modulator) (Niswender et al., 2008) inhibited RPC proliferation and promoted neuronal differentiation, whereas the knockdown of mGluR4 attenuated the effects on RPCs. Furthermore, mGluR4 activation regulated RPC behaviors by decreasing cAMP concentration, increasing the expression of PTEN and then blocking the activation of AKT signaling. Our study may reveal a brand new way to treat AMD, RP, and splitting retina diseases among others.

## Materials and Methods

### Retinal Progenitor Cell Culture

Rats used in this research were obtained from the Experimental Animal Center of the Xi’an Jiaotong University Health Science Center (Certificate No. 22-9601018). This study was carried out in accordance with the National Institutes of Health Guide for the Care and Use of Laboratory Animals (NIH Publications No. 80-23). The methods used in this study were approved by the Animal Care and Use Regulation of Xi’an Jiaotong University Health Science Center. All efforts were made to minimize animal suffering and to keep the numbers of animals used to a minimum.

P1d Sprague–Dawley (SD) rats’ eyes were dissected and their neuroretina was gently removed. Enucleated eyes were collected in pre-cooling Dulbecco’s Modified Eagle Medium: Nutrient Mixture F-12 (DMEM/F12, Gibco, United States), and the posterior retinal tissue and ciliary body were retained. The retina tissue was excised out from the retinal pigment epithelium (RPE) and dissociated into small pieces, followed by enzymolysis with 0.05% trypsin for 3 min at 37°C. Next, the tissue was mechanically dissociated with the pipette and filtrated using a 40 μm cell strainer (BD Falcon, United States), after which it was centrifuged at 1000 rpm for 3 min. After suspending and counting, cells were seeded at a density of 300,000 cells/mL in non-adherent T75 flasks with a completed medium [serum-free DMEM/F12 containing 2% B27, 1% N2, 25 μg/mL epidermal growth factor (EGF), 10 ng/mL basic fibroblast growth factor (bFGF), 1% streptomycin, 1% penicillin, and 2.5 μg/mL heparin]. All of these reagents were purchased from Gibco. Cells were then cultured in a humidified atmosphere (Sanyo, Japan) with 5% CO_2_ and 95% air at 37°C. Next, 90–150 μm of primary neurospheres (P0 cells) were observed after culturing for 3–5 days. After separating them into single cells, 50,000 cells/mL of cells were cultured in the completed medium and secondary neurospheres (P1 cells) were obtained, after which the P1 cells were used for the single-cell adhesive culture. Briefly, secondary neurospheres were dissociated into single cells using TrypLE (Invitrogen, United States). Next, single cells were cultured in poly-D-lysine (PDL)-coated dishes and plated using glass coverslips.

### Experimental Treatments

To assess the dose− and time−dependent effects of VU0155041 (mGluR4 positive allosteric selective modulator, Abcam, United Kingdom) (Betts et al., 2012), a series of VU0155041 concentration (1, 3, 5, 10, 30, and 50 μM) were added in the medium and cultured for different durations (2, 6, 12, 24, 48, and 72 h). For the mGluR4 knockdown, cells were transfected with mGluR4-targeted siRNAs or a negative control (siNC), and 30 μM VU0155041 was added in the medium after transfecting 12 h; for the negative control, the medium contained the same volume of solvent. To rule out any potential off-targets and unspecific effects of VU0155041, 10 μM LAP-4 (an orthosteric agonist for group III mGluRs, Abcam, United Kingdom) was used in this study. To detect the effect on cAMP/PTEN/AKT, 10 μM forskolin (an activator of adenylyl cyclase), 10 μM LY294002 (a highly selective Akt inhibitor) and 2 μM VO-OHpic trihydrate (VO-OH, a PTEN inhibitor) were added into the medium, respectively. For the VO-OH + VU treatment, cells were pretreated with 2 μM VO-OH, after which 30 μM VU0155041 was added to the medium after 1 h. The drugs were diluted into the culture medium, after which cells were treated by the medium, which contained a suitable concentration of drugs. In the control group, the same volume of vehicle was added into the medium. All experiments were performed in triplicate and repeated at least three times.

### Immunostaining

For immunostaining *in vivo*, the adult rats (250–300 g) were anesthetized by isoflurane and transcardially perfused with 300 mL of normal saline followed by fixing with 400 mL of 4% PFA. Next, eyeballs were post-fixed by 4% PFA at 4°C. After 7 days, the eyeballs were dehydrated in a 30% sucrose solution for 3 days at 4°C. Frozen coronal sections of 15 μm were cut using a microtome (Slee, Germany) and mounted onto glass slides for immunostaining. For immunostaining *in vitro*, cultured RPCs were fixed in 4% PFA for 30 min at room temperature, after which they were washed three times with PBS. For BrdU labeling, samples were pretreated with 2 N HCl for 30 min at 37°C, after which they were neutralized with 0.1 M borate buffer (pH8.5) for 15 min. The samples were permeabilized with 0.3% Triton X-100 (Sigma-Aldrich, St. Louis, MO, United States) for 20 min, washed 3 times with PBS, and then blocked for 2 h in a blocking buffer consisting of 5% bovine serum albumin (Sigma-Aldrich) and 5% normal goat serum (Sigma-Aldrich). The samples were incubated with the primary antibodies (Supplementary Table 1) overnight at 4°C, washed with PBS, and then incubated with suitable secondary antibodies for 2 h. Immunostaining of negative controls was carried out either by omitting the secondary antibodies or by replacing the primary antibody with block solution. Images were captured using a fluorescent microscope equipped with a digital camera (BX51 + DP71, Olympus, Japan).

### CCK-8 Assay

A Cell Counting Kit-8 (CCK-8; Sigma-Aldrich) assay was used to evaluate cell viability. RPCs were grown in poly-L-lysine-coated 96-well plates at 4000 cells/well for 24 h before the experiments. Next, cells were exposed to a series of VU0155041 concentrations (0, 0.3, 1, 10, 30, and 50 μM) for 2, 12, 24, 48, and 72 h; 0 μM VU0155041 meant that cells were just treated with the same volume of the medium. At the end of each time point, 20 μL CCK-8 was added in each well and left to incubate for 2 h. The absorbance was detected at 490 nm using a multi-microplate spectrophotometer (BioTek, United States). Triplicate parallel wells were evaluated in all tests, and the data were obtained from the average of at least three independent experiments. The results are presented as a percentage of absorbance in the control cells.

### siRNA-Mediated mGluR4 Knockdown

siRNA specific to rat mGluR4 (simGluR4-1: 5′-GCAUGUCACCAUAAUUUGCTT-3′; simGluR4-2: 5′-GGUCAUCGGCUCAUGGACATT-3′) and a scrambled siRNA negative control (siNC: 5′-CGTACGCGGAATACTTCGATT-3′) were synthesized by Genepharma (Shanghai, China). siRNA was delivered using a commercial Lipofectamine 2000 transfection reagent (Invitrogen, United States). RPCs were plated onto PLL-coated 6- or 24-well plates and cultured for 24 h before transfection. Knockdown efficiency was confirmed by Western blotting. The cells were used for further treatments at 6 h after siRNA transfection. All experiments were performed in triplicate and independently repeated at least three times.

### cAMP Assay

RPCs were grown in poly-L-lysine-coated 96-well plates and cultured for 1 day. After the transfection treatment, cells were treated with 30 μM VU0155041 or 10 μM Forskolin, with the control group medium just containing the same volume of solvent. After culturing for 3 days, the concentration of intracellular cAMP was detected using an ELISA kit (R&D Systems, United States). The absorbance was detected at 450 nm using a multi-microplate spectrophotometer (BioTek). Triplicate parallel wells were carried out, and the data were obtained from the average of at least three independent experiments.

### TUNEL Staining

To observe apoptotic cells, a TUNEL assay was carried out according to the manufacturer’s instructions (Roche Diagnostics, Switzerland). Briefly, RPCs were cultured on PDL-coated coverslips. After the treatment, cells were fixed with 4% PFA for 20 min at RT. Next, 0.1% Triton X-100 in 0.1% sodium citrate for 2 min on ice was used to permeabilize the cells. The cells were then treated with 50 μL of a TUNEL reaction mixture for 1 h at 37°C, and the nuclei were detected with DAPI (1 μg/mL). Images were captured by Olympus BX51 fluorescence microscopy with a digital camera and further processed using the Image-Pro Plus 5.0 software. Ten random fields were counted for each sample under 40X objective. Results are shown as the percentage of TUNEL-positive cells among the total number of cells (DAPI stained cells).

### Western Blot Analysis

Protein extraction of cultured RPCs was carried out using a RIPA lysis buffer (Pierce, United States) complemented with a Protease Inhibitor Cocktail (Roche, Germany). Insoluble material was removed by centrifugation at 12,000 × *g* for 10 min at 4°C. Protein concentrations were determined using the BCA method (Pierce) and adjusted in line. A total of 20–40 μg of each protein was subjected to electrophoresis on 10–12% SDS polyacrylamide gels (SDS-PAGE) and transferred to PVDF membranes. Membranes were blocked for 1 h in 5% non-fat dry milk in Tris-HCl buffer containing 0.05% Tween-20 (TBST), after which they were incubated in primary antibodies (showed in Supplementary Table 1) overnight at 4°C. After washing three times in TBST, membranes were then incubated in an HRP-conjugated secondary antibody (showed in Supplementary Table 1) for 2 h at room temperature. After being thoroughly rinsed, immunoreactive bands were detected using an enhanced chemiluminescent (ECL) substrate (Pierce) and exposed to a Fuji X-ray film (Japan). The bands were collected with a G: box gel imaging system (Syngene, United Kingdom) and analyzed with the ImageJ software (NIH). The housekeeping β-actin was used as the internal control to normalize the levels of target proteins.

### Quantification and Statistical Analysis

The TUNEL- and double immunofluorescence-stained positive cells were measured and defined using the Image-Pro Plus 5.1 software (Olympus, Japan). Significance testing was evaluated by a one-way ANOVA, followed by a Tukey’s *post hoc* test. All statistical analyses were performed using GraphPad Prism 5.0 software (San Diego, CA, United States). The data are shown as mean ± standard deviation and *P* < 0.05 was considered a statistically significant difference.

## Results

### mGluR4 Is Functionally Expressed in Rat RPCs

**FIGURE 1:**
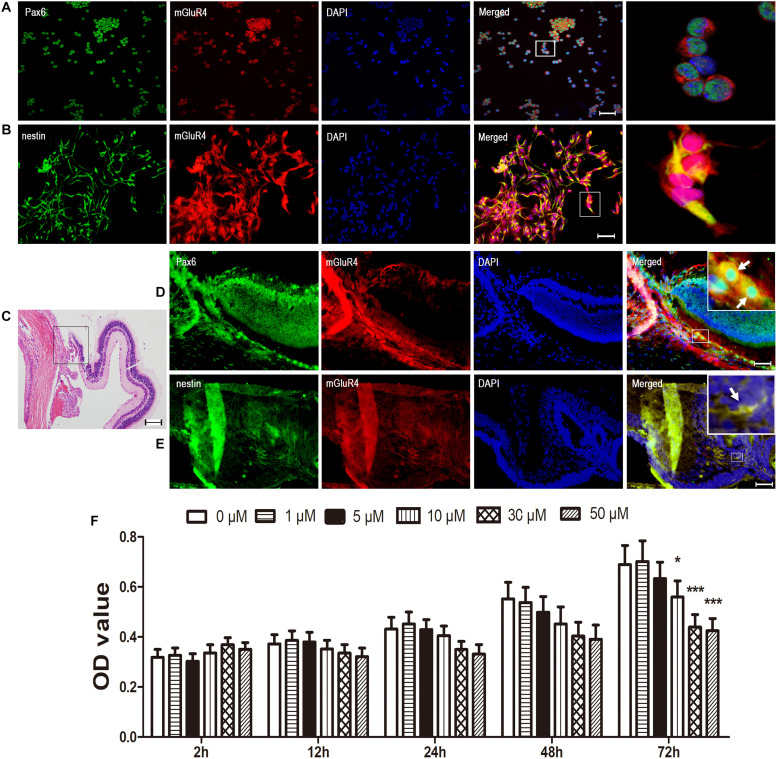
mGluR4 is functionally expressed in rat RPCs. **(A,B)** Immunofluorescent staining shows that mGluR4 (red) is co-localized with RPC marker Pax6 (green in **A**) and nestin (green in **B**) in cultured rat RPC. Scale bars in A and B are 50 μm. **(C)** Eye tissue was observed by HE staining, in the area of the ciliary body, at the retinal border as indicated by the square frame. **(D,E)** mGluR4 co-expressed with RPC-specific markers Pax6 by *in vivo* immunocytochemical double labeling, with the arrows indicating double-labeled cells; Scale bar in **(D,E)** is 100 μm. **(F)** RPCs were treated with the mGluR4 agonist VU0155041 (0, 1, 5, 10, 30, and 50 μm) for 2, 12, 24, 48, and 72 h. The RPC viability was detected by a CCK-8 assay at the end of the different treatments. Each value represents the mean ± SD of three independent experiments (*n* = 3). ^∗^*P* < 0.05, ^∗∗∗^*P* < 0.001 versus control (0 μm).

To characterize the expression of mGluR4 in RPCs, we collected rat eyeballs and cultured RPCs. Next, we examined Pax6/nestin (both of them are RPCs markers) mGluR4 by immunocytochemical double labeling, and 94.82% ± 4.43% Pax6 and 96.02% ± 7.19% nestin-positive cells co-stained with mGluR4 (Figures 1A,B). Furthermore, mGluR4 was also localized in the ciliary body, at the retinal border, and co-stained with Pax6 and nestin (Figures 1C–E). Previous research showed that the activation of mGluR4 could reduce the concentration of intracellular cAMP and affect cell viability (Byrnes et al., 2009; Zhang et al., 2019). To assess whether mGluR4 was functionally expressed in RPCs, cAMP concentrations and cell viability were detected by an ELISA and CCK-8 assay, respectively. Single-plated RPCs were treated with a different concentration of VU0155041 (0, 1, 5, 10, 30, and 50 μM) at different time points (2, 12, 24, 48, and 72 h). The cell viability was reduced by VU0155041 treatment in a dose- and time-dependent manner, as shown by a CCK-8 assay (Figure 1F). An obvious inhibitory effect was observed in 10 μM at 72 h, and with increasing concentrations, the effect was increasingly evident, reaching a peak at 50 μM. Because VU0155041 exhibited little differences between 30 and 50 μM, and VU0155041 (from 1 to 50 μM) showed no cytotoxicity in the cultured RPCs (data not shown), VU0155041 treatment at 30 μM for 3 days was applied in the following experiments. These findings strongly indicated that mGluR4 is functionally expressed in RPCs, thereby compromising cell viability.

### mGluR4 Activation Inhibits Cell Proliferation in Cultured Rat RPCs

**FIGURE 2:**
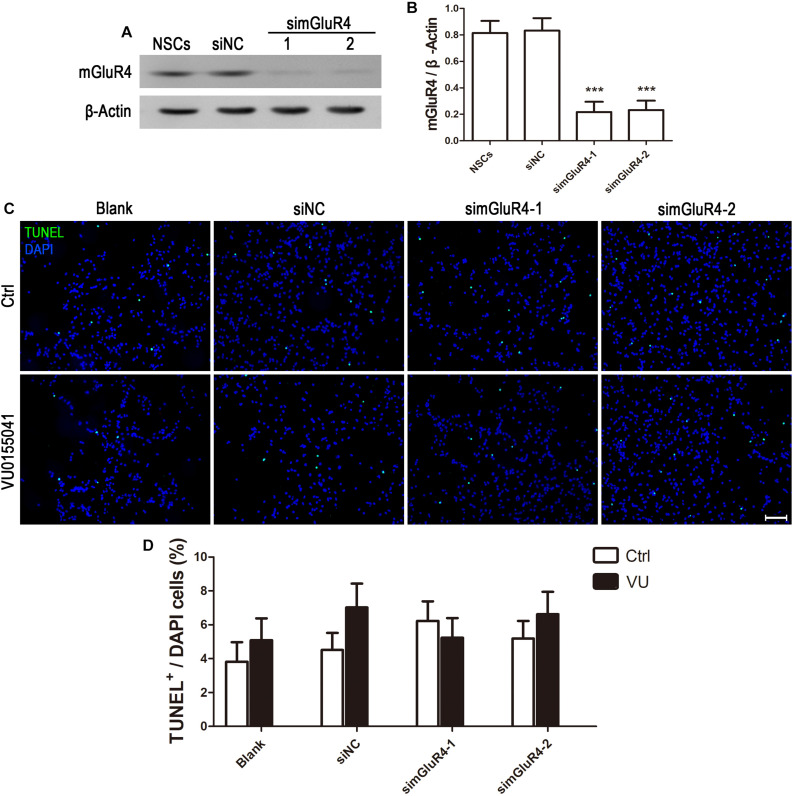
Knockdown mGluR4 does not induce cell apoptosis. **(A,B)** RPCs were transfected for 6 h with non-specific siRNAs (siNC) or mGluR4-targeted siRNA (simGluR4-1 and 2) using Lipofectamine 2000. An equal volume of medium was added to the control group, and neural stem cells (NSCs) were used as a positive control. On the second day, a Western blot analysis showed that two targeting mGluR4 siRNAs (simGluR4-1 and 2) effectively reduced mGluR4 expression. The values represent the mean ± SD of three independent experiments (*n* = 3). ^∗∗∗^*P* < 0.001 versus the siNC group. **(C,D)** 30 μM VU0155041 was added in the medium after transfecting for 12 h and cultured for 3 days. The apoptotic cells were distinguished from viable cells by TUNEL staining. Data from three independent experiments (*n* = 3) are presented as the percentage of TUNEL-positive cells in the total DAPI-stained cells.

**FIGURE 3:**
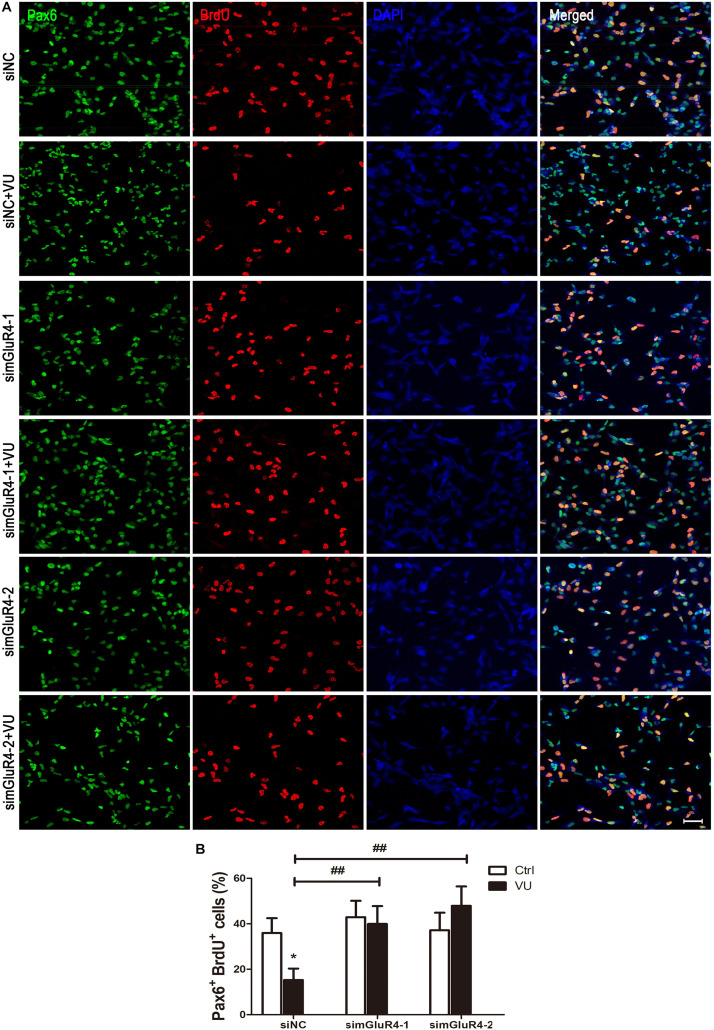
mGluR4 activation inhibits proliferation in cultured RPCs. **(A)** RPCs were transfected for 6 h by non-specific siRNA (siNC) or two mGluR4-targeted siRNAs (simGluR4-1 and simGluR4-2) using Lipofectamine 2000. After the transfection treatment, cells were incubated with 30 μM VU0155041 for 3 days, and BrdU (10 μg/mL) incorporation was used for detecting proliferation cells. **(B)** Quantitative data from three independent experiments (*n* = 3) were shown as the percentage of BrdU-positive cells in total Pax6-stained cells. Scale bar = 50 μm. ^∗^*P* < 0.05 versus control (Ctrl); ^##^*P* < 0.01 versus siNC plus VU group.

To suppress mGluR4 activity, mGluR4 was knocked down by RNA interference in this study. mGluR4 expression was detected by RT-PCR and Western blotting. The results showed that mGluR4 expression was significantly decreased after the transfection of mGluR4-targeted siRNAs (simGluR4-1 or 2) compared with both the negative control (siNC) and positive control (NSCs) (Figures 2A,B). Indeed, transfection may induce cell apoptosis, thereby affecting the cell proliferation results. TUNEL staining showed that there was no significant difference in apoptotic cells for different treatments (Figures 2C,D). This finding suggested that transfection did not cause cell death in cultured RPCs. To assess the role of mGluR4 on RPC proliferation, Pax6^+^ BrdU^+^ double-positive cells were immunostained. The staining results revealed that the treatment of siNC-transfected RPCs with 30 μM VU0155041 significantly decreased the percentage of Pax6^+^ BrdU^+^ cells. However, the knockdown of mGluR4 by transfecting mGluR4-targeted siRNAs abolished the proliferation effect of VU0155041 on RPCs (Figures 3A,B). These results strongly indicate that mGluR4 activation inhibits the proliferation of RPCs.

### mGluR4 Activation Promotes Neural Differentiation in Cultured Rat RPCs

**FIGURE 4:**
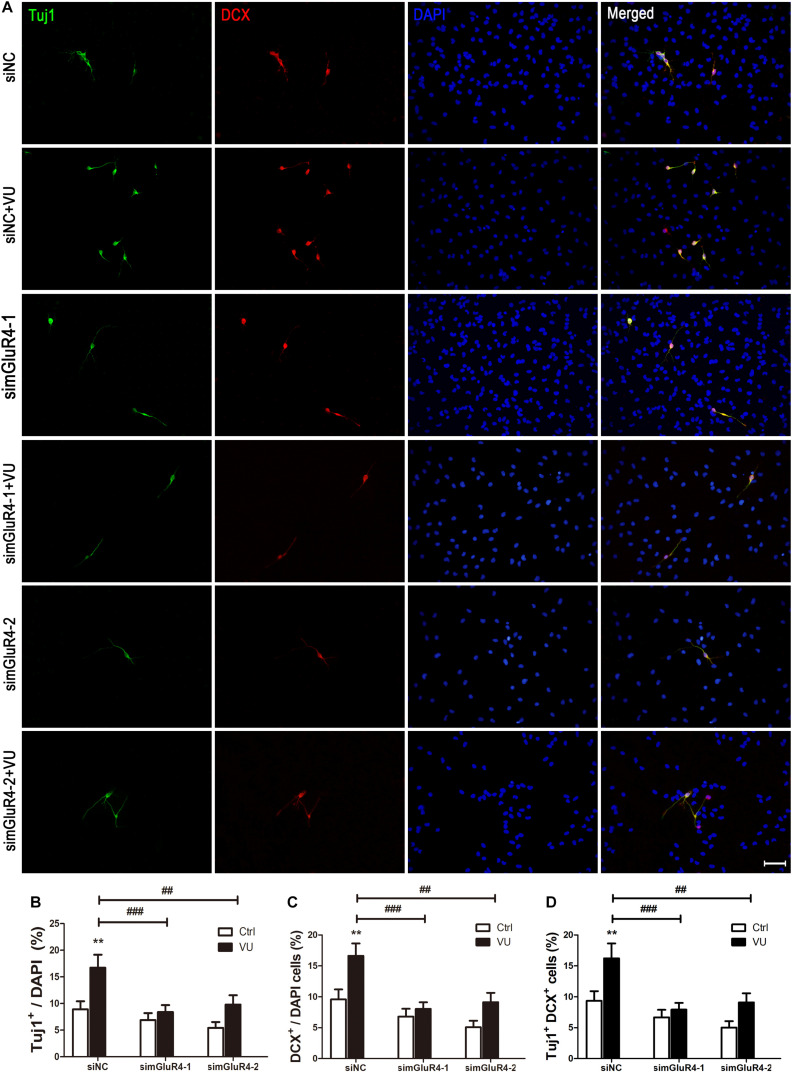
mGluR4 activation promotes neural differentiation in cultured RPCs. RPCs underwent mGluR4 knockdown and were cultured in 30 μM VU0155041 (VU) medium for 3 days. **(A)** Immunostaining was used to detect Tuj1- and DCX-positive cells. Scale bar = 50 μm. **(B,D)** The value represents the mean ± SD of three independent experiments (*n* = 3). ^∗∗^*P* < 0.01 versus control (Ctrl); ^##^*P* < 0.01, ^###^*P* < 0.001 versus siNC plus VU group.

Neuron-specific class III beta-tubulin (Tuj1) is found in the cell bodies, dendrites, axons, and axonal terminations of immature neurons, and Doublecortin (DCX) is observed in the earliest stages of neuronal development (Lee et al., 1990; Francis et al., 1999). In order to make the results more credible, both Tuj1 and DCX were used to mark immature neurons. Immunocytochemical double labeling revealed that most Tuj1-positive cells were co-stained with DCX. More importantly, compared to the control group, both Tuj1 (16.72% ± 2.44%) and DCX (16.62% ± 2.01%) were significantly increased by 16.62% ± 2.01% after exposure to VU0155041, while knocking down mGluR4 markedly abolished the effect of VU0155041 on promoting neural differentiation (Figures 4A–D).

### mGluR4 Activation Influence on the Expression of Proliferation and Differentiation Related Proteins

**FIGURE 5:**
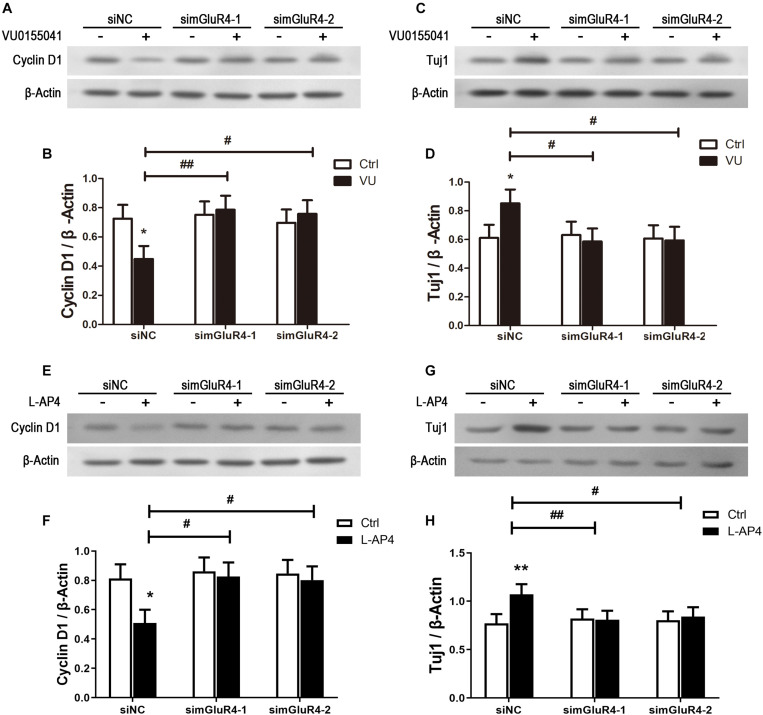
mGluR4 activation regulates the expression of differentiation- and proliferation-related proteins in cultured RPCs. RPCs were transfected with non-specific siRNA (siNC) or two mGluR4-specific siRNAs (simGluR4-1 and simGluR4-2). Next, cells were treated with 30 μM VU0155041 **(A,C)** or 10 μM L-AP4 **(E,G)** for 3 days. The changes in Cyclin D1 and Tuj1 were assessed by Western blotting. The Western blot band quantifications for the ratio of Cyclin D1 and TUj1 to β-Actin are presented. **(B,D,F,H)** The value represents the mean ± SD of three independent experiments (*n* = 3). ^∗^*P* < 0.05, ^∗∗^*P* < 0.01 versus control (Ctrl); ^#^*P* < 0.05, ^##^*P* < 0.01 versus siNC plus VU group.

To further confirm the effect of mGluR4 on proliferation and differentiation, the expression of Cyclin D1 and Tuj1 were detected by Western blotting. As excepted, the activation of mGluR4 by VU0155041 significantly inhibited Cyclin D1 expression (Figures 5A,B) and promoted Tuj1 expression (Figures 5C,D). In this study, we used VU0155041 (a positive allosteric selective modulator) to activate mGluR4. To rule out any potential off-targets and unspecific effects of VU0155041, an orthosteric agonist was used to reproduce the effect of mGluR4 on RPCs. Because no such molecule specific to mGluR4 is commercially available, LAP-4 (an orthosteric agonist for group III mGluRs) was used in the following experiment. Similar to VU0155041 treatment, 10 μM L-AP4 could significantly decrease Cyclin D1 and increased Tuj1 expression after transfecting siNC. In addition, the proliferation and differentiation effect of L-AP4 was eliminated by knocking down mGluR4 (Figures 5E–H). These phenomena may confirm the credibility of the VU0155041 results.

### mGluR4 Activation Regulates the Axis of cAMP/PTEN/AKT

**FIGURE 6:**
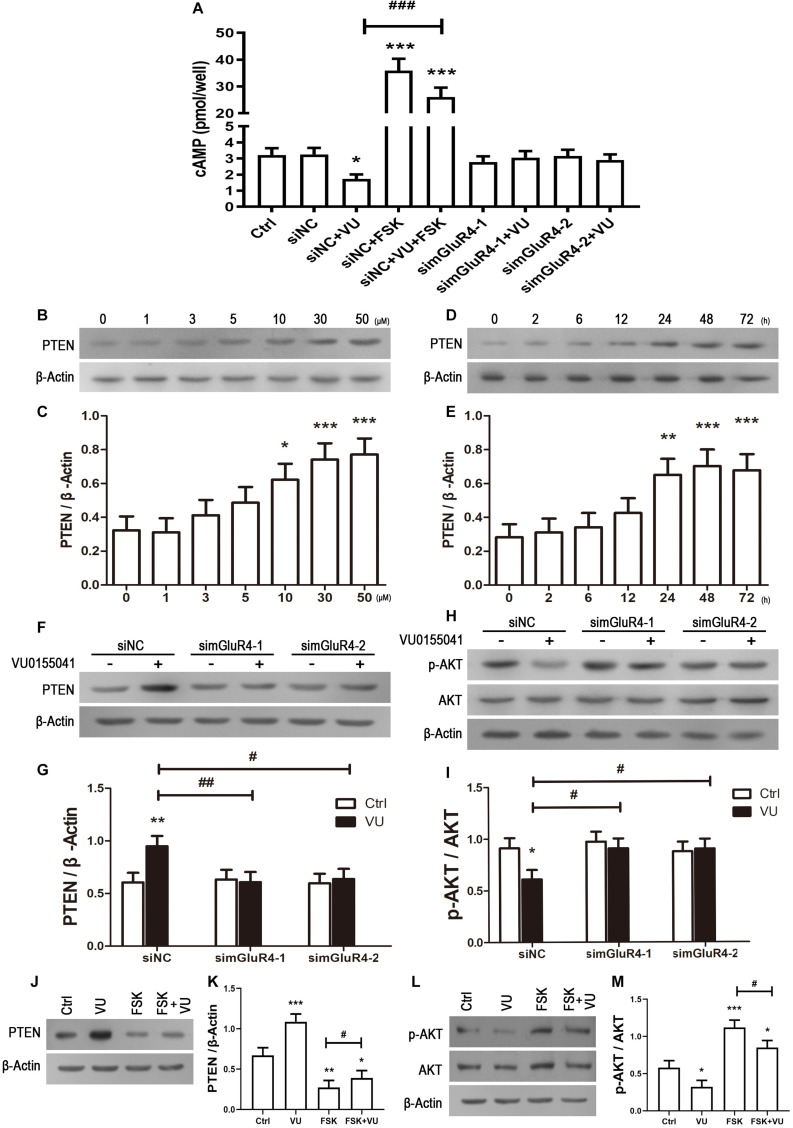
mGluR4 activation regulates the activation of the cAMP/PTEN/AKT axis. **(A)** After the transfection, cells were treated with 30 μM VU0155041 or 10 μM forskolin (FSK) for 3 days. The control group contained the same volume of solvent. Intracellular cAMP concentrations were detected by an ELISA assay. The value represents the mean ± SD of three independent experiments (*n* = 3). ^∗^*P* < 0.05, ^∗∗∗^*P* < 0.001 versus siNC group; ^###^*P* < 0.001 versus siNC plus VU group. To detect the effects of mGluR4 on PTEN, RPCs were treated with different concentrations of VU0155041 (1, 3, 5, 10, 30, and 50 μM) for 72 h or 30 μM VU015541 at a series of time points (2, 6, 12, 24, 48, and 72 h). **(B,D)** The representative WB band illustrates the ratio of PTEN to β-Actin after treatments. **(C,E)** The value represents the mean ± SD of three independent experiments (*n* = 3). ^∗^*P* < 0.05, ^∗∗^*P* < 0.01, ^∗∗∗^*P* < 0.001 versus 0 group. mGluR4 expression was knocked down using siRNAs, after which cells were treated by 30 μM VU015541 for 3 days. **(F,H)** WB band quantifications of the ratio of PTEN to β-Actin and p-AKT/AKT are presented. **(G,I)** The value represents the mean ± SD of three independent experiments (*n* = 3). ^∗^*P* < 0.05, ^∗∗^*P* < 0.01, versus control (Ctrl); ^#^*P* < 0.05, ^##^*P* < 0.01 versus siNC plus VU group. Incubation with 30 μM VU0155041 (VU), 10 μM FSK, or forskolin plus VU0155041 (FSK + VU) for 3 days. **(J,L)** The WB band quantification for the ratio of PTEN to β-Actin and p-AKT/AKT is presented. **(J,L)** The value represents the mean ± SD of three independent experiments (*n* = 3). ^∗^*P* < 0.05, ^∗∗^*P* < 0.01, ^∗∗∗^*P* < 0.001 versus control (Ctrl); ^#^*P* < 0.05 versus FSK group.

**FIGURE 7:**
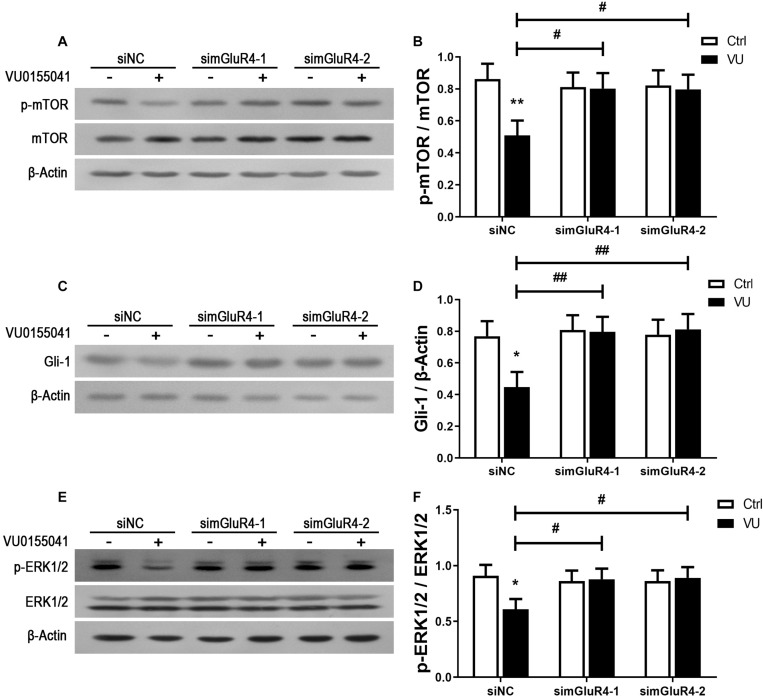
mGluR4 activation suppresses the mTOR, Shh, and MAPK signaling pathways. RPCs were transfected for 6 h by non-specific siRNA (siNC) or two mGluR4-targeted siRNAs (simGluR4-1 and simGluR4-2) using Lipofectamine 2000. After the transfection treatment, cells were incubated with 30 μM VU0155041 for 3 days. The WB band quantification of the ratio of p-mTOR/mTOR **(A)**, Gli-1 to β-Actin **(C)**, and p-ERK1/2/ERK1/2 **(E)** is presented. **(B,D,F)** The value represents the mean ± SD of three independent experiments (*n* = 3). ^∗^*P* < 0.05, ^∗∗^*P* < 0.01 versus control (Ctrl); ^#^*P* < 0.05, ^##^*P* < 0.01 versus siNC plus VU group.

In searching for a transduction pathway that could mediate the biological effects of mGluR4 on RPCs, we first focused on the inhibition of adenylyl cyclase, which is the canonical pathway associated with mGlu4 receptor activation (Canudas et al., 2004; Zhang et al., 2015, 2018, 2020). VU0155041 significantly decreased cAMP formation after transfecting siNC, and knockdown mGluR4 abolished the effect of VU0155041. Interestingly, VU0155041 significantly reduced the increase in cAMP formation induced by forskolin (Figure 6A). This suggests that mGluR4 activation could decrease intracellular cAMP concentration. Previous studies showed that the PTEN/AKT pathway was involved in processes such as differentiation, proliferation, and survival (Yu and Cui, 2016). Moreover, our earlier study has demonstrated that mGluR4 activation decreased Akt phosphorylation levels (Zhang et al., 2019). Herein, we assess PTEN and AKT phosphorylation by Western blotting exposure to VU0155041. First, RPCs were manipulated with a series of VU0155041 concentrations (0, 1, 3, 5, 10, 30, and 50 μM) for 72 h or treated with 30 μM VU015541 at different time points (2, 6, 12, 24, 48, and 72 h). Results showed that mGluR4 activation increased PTEN expression in a dose- and time-dependent manner (Figures 6B–E). More importantly, the knockdown of mGluR4 abolished the effects of VU0155041 on PTEN expression (Figures 6F,G). Next, we assessed the activation of AKT, which is downstream of PTEN. An AKT phosphorylation assay showed that VU0155041 inhibited AKT activation, while mGluR4 inhibition by mGluR4-targeted siRNAs eliminated the effects of VU0155041, indicating that the activation of mGluR4 blocked the phosphorylation levels of AKT in cultured rat RPCs (Figures 6H,I). In order to prove that mGluR4 regulates PTEN and AKT through cAMP, forskolin was used to block the effect of VU0155041. As expected, forskolin eliminated the effect of VU0155041 on PTEN expression (Figures 6J,K) and AKT activation (Figures 6L,M). Since it is generally known that the PTEN/AKT signaling pathway cross-talks with other signaling pathways (including mTOR, Sonic Hedgehog, and MAPK), we further assessed the expression of key regulatory proteins in these signaling pathways by Western blotting. Results showed that the activation of mGluR4 with VU0155041 significantly decreased the levels of p−mTOR, Gli-1, and p−ERK1/2, and that knockdown of mGluR4 markedly abolished the effects of VU0155041 (Figures 7A–F).

### mGluR4 Regulates RPCs Proliferation and Differentiation via cAMP/PTEN/AKT Signal Pathway

**FIGURE 8:**
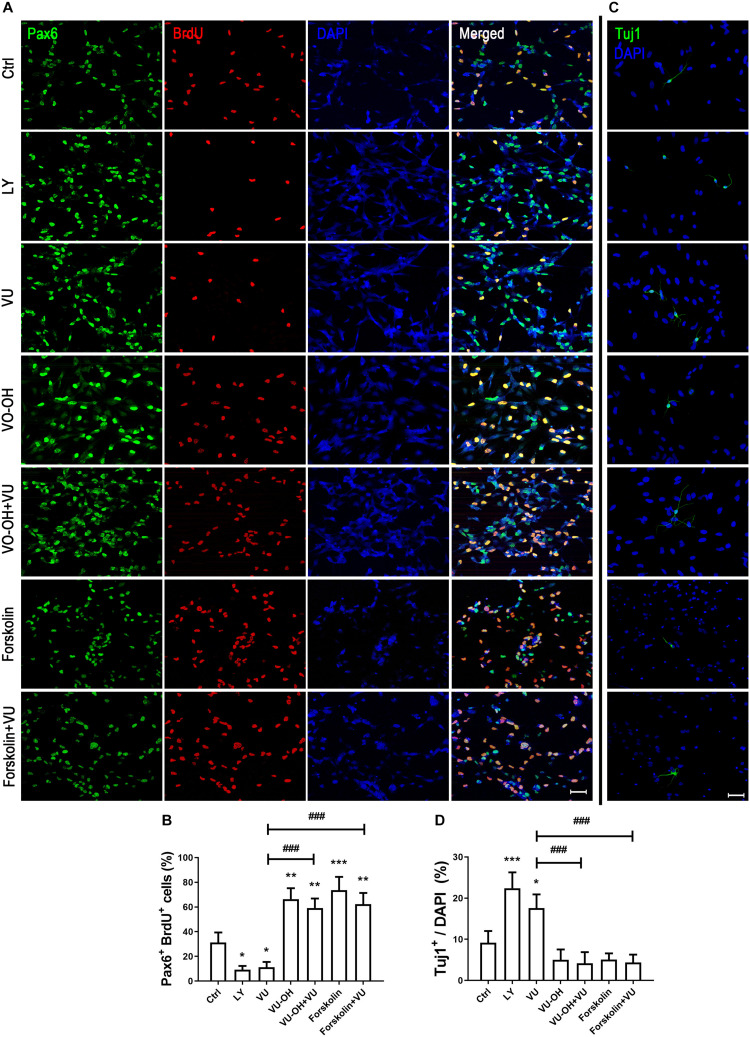
cAMP/PTEN/AKT pathway is closely involved in regulating the effect of mGluR4 in cultured RPCs. RPCs were treated with a vehicle, LY294002 (LY, 10 μM), VU0155041 (VU, 30 μM), VO-OH (5 μM), VU + VO-OH, Forskolin, or Forskolin + VU for 3 days. **(A)** Cell proliferation was analyzed by BrdU incorporation. Scale bar = 50 μm. **(C)** Neural differentiation was detected by immunostaining using Tuj1. Scale bar = 50 μm. **(B,D)** The value represents the mean ± SD of three independent experiments (*n* = 3). ^∗^*P* < 0.05, ^∗∗^*P* < 0.01, and ^∗∗∗^*P* < 0.001 versus control (Ctrl); ^###^*P* < 0.001 versus VU group.

To further explain whether the function of mGluR4 in RPCs may be regulated by the PTEN/AKT signaling pathway, we detected Pax6^+^ BrdU^+^ double-positive cells and Tuj1^+^ cells in the presence of VU0155041, cAMP activator, Akt, and PTEN inhibitors. The results showed that both LY294002 (10 μM, a highly selective Akt inhibitor) and VU0155041 significantly decreased the number of Pax6^+^ BrdU^+^ cells and increased Tuj1-positive cells. Interestingly, both the PTEN inhibitor (2 μM, VO-OH) and 10 μM Forskolin markedly attenuated the proliferation and differentiation effect of VU0155041 (Figures 8A–D). These results indicate that mGluR4 activation may influence proliferation and differentiation in cultured RPCs by regulating the cAMP/PTEN/AKT signaling pathway.

## Discussion

As a specific type of G protein-coupled receptor, metabotropic glutamate (mGlu) receptors are generally considered neurotransmitter receptors because they respond to synaptic glutamate and are involved in the regulation of synaptic plasticity (Melchiorri et al., 2007). However, evidence is mounting that mGluRs are located in and participate in regulating cell development, such as in embryonic stem cells and NSCs (Melchiorri et al., 2007; Berg et al., 2013). The activation of group 2 mGluR reduces basal levels of apoptosis and increases neural precursor proliferation (Brazel et al., 2005). mGluR6 mutant cannot bring about a complete differentiation of RPCs into bipolar neurons (Zahir et al., 2005). mGluR7 promotes the proliferation and differentiation of neural progenitor cells (Tian et al., 2010). mGluR4 could prevent oxidative stress-induced NSC death and involves the regulation of NSC behaviors (Maj et al., 2003; Zhang et al., 2015, 2020). mGluR5 activation promotes human NSC and improves the expression of Cyclin D1 by regulating the activation of MAPK signaling pathways (Zhao et al., 2011). Even though the research on mGluRs in RPCs is rather scarce, our previous study showed that mGluR5 could promote the proliferation of RPCs while activating the MAPK and PI-3-K pathways (Zhang et al., 2016). In this study, we observed that mGluR4 had an opposite effect on cultured rat RPCs. mGluR4 activation suppressed the proliferation and promoted the neuronal differentiation of RPCs by inhibiting PI-3-K pathway activation. These phenomena indicate that mGluR4/5 may be defined as a molecular switch that regulates RPC proliferation. Moreover, these studies may also point to a novel regulatory site in RPCs, in which mGluRs can be considered novel targets for ocular disease.

As a novel activator of mGluR4, VU0155041 binds to the allosteric site and positively cooperates with orthostatic agonists (like glutamate or L-AP4). Moreover, a characteristic of VU0155041 is that it has significant intrinsic agonist activity, which was not attenuated by the antagonist LY341495 (Niswender et al., 2008; Rovira et al., 2015). Previous research had demonstrated that VU0155041 could specifically activate mGluR4 without potentiating or abating other mGluR subtypes (Niswender and Conn, 2010). Therefore, VU0155041 may represent an innovative target for regulating cell function in different cell types (Betts et al., 2012; Guimaraes-Souza and Calaza, 2012; Zhang et al., 2018). Although the EC50 was 798 nM, revealing a heterogeneous mGluR4 expression system in humans, previous investigations have reported that the effective concentrations of VU0155041 in cultured mouse cortical neurons were 10 and 30 μM, and that VU0155041 did not exhibit any obvious binding activity with off-target mGluR subtypes (Domin et al., 2016). Our previous study also revealed that the effective concentration of the compound was 30 μM in terms of regulating cell proliferation, differentiation, and survival (Zhang et al., 2015, 2019, 2020). Due to the lack of knowledge with regard to the expression profiles of other mGluR subtypes in RPCs, we are unable to explore the possible off-target effects of VU0155041. We also cannot confirm the desensitizing effect on mGluR4 in response to chronic exposure to VU0155041. However, similar results were found for LAP-4 (an orthosteric agonist for group III mGluRs) with regard to the proliferation and neuronal differentiation of RPCs. These results may point to the credibility of the VU0155041 results. Based on these phenomena, future intensive investigations should be carried out in overexpression/knockdown models to resolve these key problems and further probe the possible application of VU0155041 in the treatment of RPCs.

**FIGURE 9:**
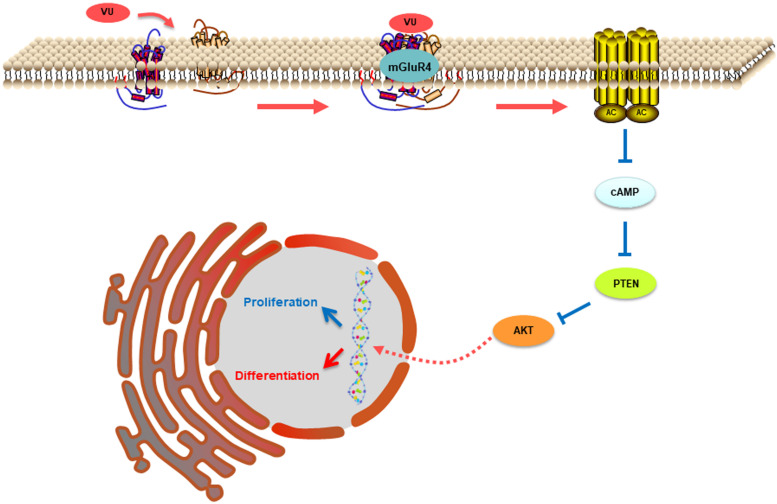
This illustration depicts the mechanisms by which mGluR4 activation regulates proliferation and differentiation in retinal progenitor cells. The intracellular cAMP levels are decreased due to mGluR4 activation by glutamate or an agonist. The inhibitory effect of cAMP on PTEN is attenuated; the low concentration results in a relative increase in PTEN expression. PTEN can decrease Akt phosphorylation, eventually influencing proliferation and neuronal differentiation.

In this study, mGluR4 activation promoted the expression of PTEN in RPCs in a dose- and time-dependent manner. Furthermore, the knockdown of mGluR4 could eliminate the effect of VU0155041 on PTEN expression. These results demonstrated that the activation of mGluR4 promoted intracellular PTEN expression. As an important signaling molecule, the regulation of PTEN expression in RPCs is crucial to control the balance of cell proliferation, survival, and differentiation. Previous study showed that neurogenesis came earlier in RPCs which lacked the PTEN gene and resulted in their premature depletion in the mature retina. This is because the Notch intracellular domain fails to form a transcription activator complex in PTEN-deficient RPCs (Jo et al., 2012). Although it remains unknown to this day whether PTEN is involved in the regulation of mGluR4 in stem cell development, some studies have showed that PTEN expression was negatively modulated by cAMP (Duan et al., 2015). Our study showed that intracellular cAMP levels were decreased by activating mGluR4. Therefore, it is reasonable to assume that mGluR4 activation decreased cAMP concentrations and in turn promoted PTEN expression in RPCs. As a phosphatase protein, PTEN acts as a phosphatase to dephosphorylate the inositol ring in PIP3, leading to a PIP2 product, and finally inhibiting the PI-3-K signaling pathway (Milella et al., 2015). Our results unambiguously indicated that the activation of mGluR4 promoted PTEN expression and inhibited Akt phosphorylation. Moreover, the effects of VU0155041 on RPCs could be abolished by an adenylyl cyclase activator and a PTEN-selective inhibitor. In contrast, LY294002 had similar effects to VU0155041, promoting neural differentiation and inhibiting proliferation. In light of all of the results, we propose that mGluR4 activation involves the regulation of rat RPC proliferation and neuronal differentiation via the cAMP/PTEN/AKT signaling axis. The activation of mGluR4 by glutamate, agonist, or allosteric modulator inhibits adenylyl cyclase activity, reducing intracellular levels of cAMP. Accompanied by decreasing cAMP, the expression of PTEN is increased and results in the blocking of phosphorylation levels of Akt, eventually suppressing proliferation while promoting neural differentiation (Figure 9).

The PI-3-K signaling pathway plays a vital role in regulating cell differentiation, proliferation, and tumorigenesis in mammals (Maiese, 2016). As known, the effect of PI-3-K signaling is not an isolated process. Instead, the biological functions of Akt play a key role through their cross-talk with other signaling pathways, including Wnt (McCubrey et al., 2014), Sonic Hedgehog (Roy et al., 2014), mTOR (Janku et al., 2014), and MAPK (Cakir and Grossman, 2009). The protein kinase cascades are generally detected in the context of cross-talk between different signaling pathways, whereby influencing one kinase cascade leads to a change in the other’s activity (Hausenloy et al., 2004). For example, the activation of Akt can inhibit RAF1 expression, resulting in reduced phosphorylation levels of ERK1/2 and inhibition of the activation of MAPK signaling pathways (Pandit et al., 2007). In this study, we observed that VU0155041 also impacted the activation of many signaling pathways, including mTOR, Sonic Hedgehog, and MAPK (Figures 7A–F). Based on the above data, it is clear that these signaling pathways form an intersecting biochemical network that modulates cell behaviors through cascade signal amplification, ultimately resulting in many biological effects including neuronal differentiation and proliferation. Further studies will be warranted to better assess the cross-talk of the PI-3-K and other pathways in the context of the proliferation and differentiation induced by mGluR4. Whether other signal pathways mediate the above-mentioned proliferation mechanism remains to be assessed.

In this study, we reported that the pharmacological activation of mGluR4, one of the group III mGluRs, inhibits RPC proliferation while decreasing the number of Pax6^+^ BrdU^+^ cells that subsequently enter neuronal differentiating progression, as indicated by the increased number of neuronal lineage-specific marker Tuj1^+^ DCX^+^ cells. Moreover, the effect of mGluR4 would seem to be involved in decreasing cAMP concentration, inhibiting PTEN expression, and subsequently suppressing AKT phosphorylation. This study provides evidence that mGluR4 is involved in the regulation of cultured rat RPC proliferation and differentiation. However, the mechanisms by which mGluR4 regulates the PTEN/AKT signal pathway in the context of the modulation of cell behaviors remains open and warrants further investigation.

## Data Availability Statement

The datasets generated for this study are available on request to the corresponding author.

## Ethics Statement

The animal study was reviewed and approved by the Xi’an Jiaotong University Health Science Center Comments of the laboratory animal care committee.

## Author Contributions

ZZ, XZ, XC, and YoL designed the experiments. XZ, HL, XC, and YaL, supervised the research. YiL, YLu, and LC performed animal breeding. ZZ, XZ, BH, BM, and XL performed most of the other experiments. ZZ prepared the manuscript drafts. KZ, XZ, HL, and YoL edited the manuscript. All authors contributed to the article and approved the submitted version.

## Conflict of Interest

The authors declare that the research was conducted in the absence of any commercial or financial relationships that could be construed as a potential conflict of interest.

## Abbreviations

AMD: age-related macular degeneration
CCK8: Cell Counting Kit-8
mGluR: metabotropic glutamate receptor
mGluR4: type 4 metabotropic glutamate receptor
NSCs: neural stem cells
RP: retinitis pigmentosa
RPCs: retinal progenitor cells
RPE: Retinal pigment epithelial.
