# Supplementary material for: Activation of Type 4 Metabotropic Glutamate Receptor Regulates Proliferation and Neuronal Differentiation in a Cultured Rat Retinal Progenitor Cell Through the Suppression of the cAMP/PTEN/AKT Pathway
Source: Front Mol Neurosci. 2020 Aug 20;13:141. doi: 10.3389/fnmol.2020.00141 (PMC7469868; doi:10.3389/fnmol.2020.00141)
Supplement: Supplementary file 1 [file Table_1.DOCX]

| Table I Antibodies used during the study | | | | |
| --- | --- | --- | --- | --- |
| Antigen | Source and host species | Concentration | Catalog No. | RRID |
| anti-mGluR4 | Abcam, rabbit polyclonal antibody | 1:200 | ab53088 | AB_881158 |
| anti-BrdU | Abcam, sheep polyclonal antibody | 1:200 | ab1893 | AB_302659 |
| anti-PAX6 | Abcam, mouse monoclonal antibody | 1:100 | Ab78545 | AB_1566562 |
| anti-DCX | Abcam, rabbit polyclonal antibody | 1:1000 | ab18723 | AB_732011 |
| anti-nestin | Milliproe, mouse monoclonal antibody | 1:200 | MAB5326 | AB_11211837 |
| anti-Tuj1 | Milliproe, mouse monoclonal antibody | 1:200 (IF)  1:500 (WB) | MAB1637 | AB_2210524 |
| anti-Cyclin D1 | Cell Signaling Technology, rabbit polyclonal antibody | 1:1000 | 2922 | AB_2228523 |
| anti-phospho-AKT | Cell Signaling Technology, rabbit monoclonal antibody | 1:1000 | 4060 | AB_2315049 |
| anti-AKT | Cell Signaling Technology, rabbit polyclonal antibody | 1:1000 | 9272 | AB_329827 |
| anti-PTEN | Cell Signaling Technology, rabbit polyclonal antibody | 1:1000 | 9552 | AB_10694066 |
| anti-β-actin | Sigma-Aldrich, mouse monoclonal antibody | 1:5000 | A1978 | AB_476692 |
| anti-mouse IgG (H+L) secondary antibody, Alexa Fluor 488 | Invitrogen, goat polyclonal antibody | 1:500 | A-11001 | AB_2534069 |
| anti-sheep IgG (H+L) secondary antibody, Alexa Fluor 594 | Invitrogen, donkey polyclonal antibody | 1:500 | A-11016 | AB_2534083 |
| anti-rabbit IgG (H+L) secondary antibody, Alexa Fluor 594 | Invitrogen, goat polyclonal antibody | 1:500 | A-11012 | AB_141359 |
| HRP-conjugated anti-Mouse IgG | Sigma-Aldrich, goat polyclonal antibody | 1:10000 | AP130P | AB_91266 |
| HRP-conjugated anti-rabbit IgG | Sigma-Aldrich, goat polyclonal antibody | 1:10000 | AP307P | AB_92641 |
